# Prozac in the water: Chronic fluoxetine exposure and predation risk interact to shape behaviors in an estuarine crab

**DOI:** 10.1002/ece3.3453

**Published:** 2017-09-30

**Authors:** Joseph R. Peters, Elise F. Granek, Catherine E. de Rivera, Matthew Rollins

**Affiliations:** ^1^ Ecology, Evolution, & Marine Biology University of California Santa Barbara Santa Barbara CA USA; ^2^ Environmental Science & Management Portland State University Portland OR USA

**Keywords:** emerging contaminants, estuaries, fluoxetine, multiple stressors, pharmaceuticals, predation risk, trophic interactions, water quality

## Abstract

Predators exert considerable top‐down pressure on ecosystems by directly consuming prey or indirectly influencing their foraging behaviors and habitat use. Prey is, therefore, forced to balance predation risk with resource reward. A growing list of anthropogenic stressors such as rising temperatures and ocean acidification has been shown to influence prey risk behaviors and subsequently alter important ecosystem processes. Yet, limited attention has been paid to the effects of chronic pharmaceutical exposure on risk behavior or as an ecological stressor, despite widespread detection and persistence of these contaminants in aquatic environments. In the laboratory, we simulated estuarine conditions of the shore crab, *Hemigrapsus oregonensis,* and investigated whether chronic exposure (60 days) to field‐detected concentrations (0, 3, and 30 ng/L) of the antidepressant fluoxetine affected diurnal and nocturnal risk behaviors in the presence of a predator, *Cancer productus*. We found that exposure to fluoxetine influenced both diurnal and nocturnal prey risk behaviors by increasing foraging and locomotor activity in the presence of predators, particularly during the day when these crabs normally stay hidden. Crabs exposed to fluoxetine were also more aggressive, with a higher frequency of agonistic interactions and increased mortality due to conflicts with conspecifics. These results suggest that exposure to field‐detected concentrations of fluoxetine may alter the trade‐off between resource acquisition and predation risk among crabs in estuaries. This fills an important data gap, highlighting how intra‐ and interspecific behaviors are altered by exposure to field concentrations of pharmaceuticals; such data more explicitly identify potential ecological impacts of emerging contaminants on aquatic ecosystems and can aid water quality management.

## 1 INTRODUCTION

Animal behaviors are rooted within their realized niche: individuals modify their behaviors to balance risks (e.g., predation, competition) with rewards (e.g., access to resources; De Roos, Persson, & McCauley, 2003; Brown & Kotler, 2004). Active behaviors such as foraging, moving about, or interactions with conspecifics are important for prey survival but are considered risky when there is an immediate threat of predation (Lima & Dill, 1990; Preisser, Orrock, & Schmitz, 2007). Observable patterns in prey risk behaviors often depend on the spatial or temporal context of their predator (Morgan, Spilseth, Page, Brooks, & Grosholz, 2006; Snell‐Rood, 2013), as there are certain areas and times that are more dangerous due to predator activity. Prey often shape their foraging behaviors so they are out of sync with their predators (e.g., remaining hidden during the day/emerging at night), thereby reducing their chances of an encounter (Lima & Dill, 1990). Within a species, there is also considerable variability in individual risk behaviors due to differences in size and sex (Blanckenhorn, 2005) as those with better defenses (e.g., claws, armor) are often bolder and take greater risk than those without. In social groups, better‐defended individuals often take a position of dominance and exhibit more agonistic behaviors, fighting with conspecifics for access to mates and other resources (Drews, 1993; Sneddon, Taylor, Huntingford, & Watson, 2000). Prey risk behaviors are thus shaped by both intra‐ and interspecific interactions where an individual's survival is enhanced by taking risks at the right place and time.

While predator–prey behavior dynamics are regulated by a combination of abiotic and biotic factors (Chase, Biro, Ryberg, & Smith, 2009; Grabowski, 2004), typically the limiting physical factors (e.g., temperature, salinity, and photoperiod) are naturally occurring. Interactions between multiple species further restrict niches and may be modulated by such physical conditions, as famously demonstrated by Connell (1961) where both competition and physical stressors limit barnacle distribution in the rocky intertidal. However, a growing list of anthropogenic stressors has been shown to alter normal animal behaviors, leading to reduced fitness, changes in population structure, and modification of ecosystem function (Barros, 2001; Dodd, Grabowski, Piehler, Westfield, & Ries, 2015; Fahrig, 2007; Frid & Dill, 2002). Fisheries have historically targeted large predators and directly modified community processes through release from predation pressure (Catano et al., 2016). Ocean acidification alters the development of larval fishes, disrupting their ability to detect predator cues, leading to increased mortality (Munday et al., 2009). Exposure to heavy metals, pesticides, and other legacy contaminants has been shown to affect animal behaviors by altering habitat preference, shifting migration patterns, or increasing negative species interactions (Fleeger, Carman, & Nisbet, 2003; Fukunaga, Anderson, Webster‐Brown, & Ford, 2010; Khoury, Powers, Patnaik, & Wallace, 2009; Menone et al., 2006). These anthropogenic impacts have been shown to limit the realized niche of an organism beyond what are traditionally considered natural restrictions.

Much less studied are the effects of pharmaceuticals and other emerging contaminants as stressors and how they alter animal behavior, despite frequent detections of these compounds in aquatic environments (Boxall et al., 2012; Brausch, Connors, Brooks, & Rand, 2012; Gaw, Thomas, & Hutchinson, 2014). Pharmaceutical compounds and their derivatives regularly enter estuaries and near‐shore coastal ecosystems via transport of contaminated surface and groundwater runoff, suspended river sediments, and untreated sewage effluent (Bringolf et al., 2010; Khairy, Weinstein, & Lohmann, 2014; Metcalfe et al., 2010). These compounds are designed to illicit biological responses as medical drugs and could have considerable effects on organism health, despite detections at low concentrations (Ankley, Brooks, Huggett, & Sumpter, 2007; Seiler, 2002). Prolonged studies on marine organisms at environmentally relevant concentrations are lacking (Gaw et al., 2014; Prichard & Granek, 2016) and most pharmaceutical exposure studies are rooted in ecotoxicological methodology with adverse outcomes determined at the cellular or subcellular level (Boxall et al., 2012). Exposure studies that assess effects of pharmaceuticals on whole‐organism metrics, and multiorganism or community‐level interactions are needed to improve our understanding of their effects on natural systems (Fleeger et al., 2003; Brooks, Huggett, & Boxall, 2009; Corcoran, Winter, & Tyler, 2010; Gaw et al. 2014).

Selective serotonin reuptake inhibitor (SSRI) antidepressants such as fluoxetine hydrochloride (Prozac^®^) are among the more prevalent categories of pharmaceuticals detected in the marine environment (Kreke & Dietrich, 2008; Vasskog et al., 2008; Brodin et al., 2014; Gaw et al., 2014). SSRIs have been developed to delay the reuptake of serotonin, moderating neurotransmission in the human brain. In crustaceans, serotonin is known to affect behaviors through stimulating the release of hyperglycemic, neuro‐depressing, molt‐inhibiting, and gonad‐stimulating hormones (Fong & Ford, 2014). McPhee and Wilkens (1989) found that *Carcinus maenas* crabs injected with serotonin increased their activity levels during the day, whereas normally they are photonegative. In the same crab species, 120 μg/L of fluoxetine significantly altered locomotor behaviors (Mesquita, Guilhermino, & Guimaraes, 2011). Several other studies have demonstrated that fluoxetine leads to adverse physiological and behavioral outcomes in aquatic organisms that could alter their functional roles within the community (Bossus, Guler, Short, Morrison, & Ford, 2014; Chen, Zha, Yuan, & Wang, 2015; Dzieweczynski & Hebert, 2012; Munari, Marin, & Matozzo, 2014; Peters & Granek, 2016; Schultz et al., 2011).

Relatively, few studies have assessed how pharmaceuticals affect interspecific interactions such as predator–prey dynamics (see Brodin et al., 2014; Gaw et al., 2014; Prichard & Granek, 2016 for reviews). Yet, several studies have hypothesized by stimulating activity levels, those contaminants would increase risk of predation and mortality (Brodin et al., 2014; Corcoran et al., 2010; Hazelton et al., 2014; Schultz et al., 2011). To address this data gap, we conducted a laboratory study to assess how predator presence and prolonged exposure to the pharmaceutical contaminant fluoxetine interact to shape risk behaviors among the shore crab, *Hemigrapsus oregonensis*. Fluoxetine has been frequently detected in coastal areas at low concentrations (0.03–300 ng/L; Kreke & Dietrich, 2008; Vasskog et al., 2008) and is considered toxic to fish and marine invertebrates at high concentrations (Brooks et al., 2003). We were interested in the role of fluoxetine as a persistent ecological stressor in estuaries where sublethal concentrations between 3 and 30 ng/L are commonly detected (Kreke & Dietrich, 2008; Vasskog et al., 2008). We conducted a series of diurnal and nocturnal behavioral trials over 9 weeks to assess whether fluoxetine exposure altered risk behaviors of *H. oregonensis* in response to a predator, the red rock crab *Cancer productus*. We hypothesized that prolonged exposure to these concentrations of fluoxetine would increase *H. oregonensis* foraging and locomotor activity, resulting in increased predation risk. We also hypothesized that alterations in risk behaviors due to fluoxetine exposure would increase active behaviors during the day when crabs are typically withdrawn or buried. Lastly, we hypothesized that fluoxetine exposure would alter the agonistic behaviors among crabs of different sex and size classes. To our knowledge, our study is the first to assess how pharmaceutical contaminants affect risk behaviors in marine animals.

## 2 MATERIALS AND METHODS

### 2.1 Study animals and experimental setup

**Figure 1:**
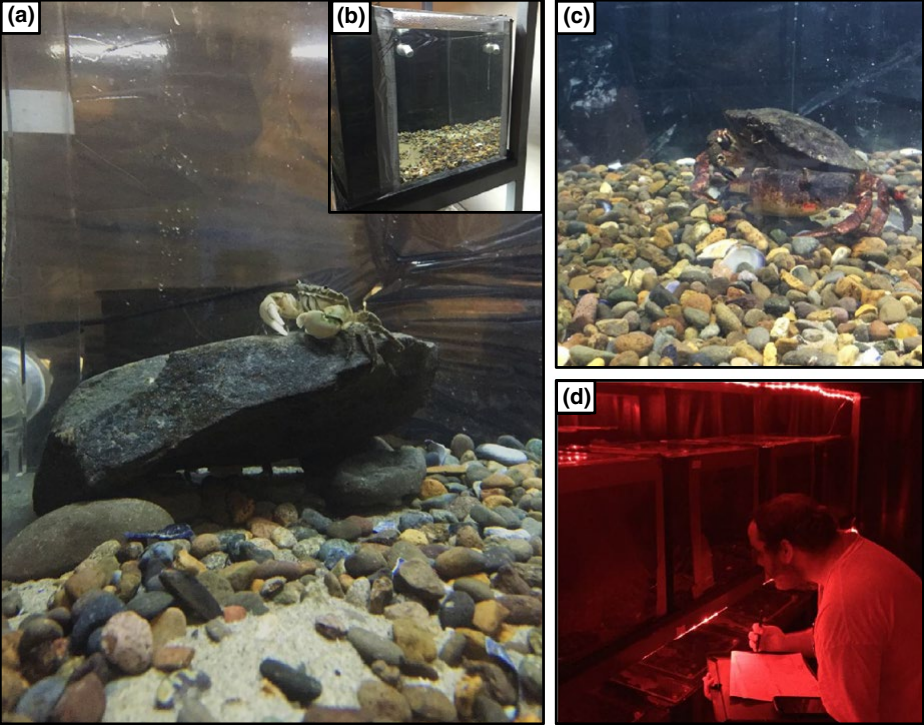
Pictures of (a) a *Hemigrapsus oregonensis* in the aquarium habitat, (b) example of the tank set up with sides blacked out, (c) addition of *Cancer productus* during predator trials, and (d) an observer recording crab behavior during a night trial

The Oregon shore crab, *H. oregonensis* (Dana, 1851; Figure 1a), is a small intertidal crab belonging to the family Grapsidae and is one of the most common species inhabiting estuarine shorelines between Resurrection Bay, Alaska, USA, and Bahia de Todos Santos, Baja California, Mexico (Lindberg, 1980). This crab forages mostly at night, primarily eating diatoms and green algae, but also eating carrion and other meat, if available (Lindberg, 1980). *Hemigrapsus oregonensis* spends most of its time on, beneath, or near rocks in gravel and fine sediment substrate. To escape predators, *H. oregonensis* often quickly burrows in sediment or hides beneath rocks; it also relies on camouflage while remaining motionless (Lindberg, 1980).

The red rock crab, *Cancer productus* (Randall, 1839; Figure 1c), is one of several *Cancer* species that inhabit the Pacific Coast of North America, occupying a similar range as *H. oregonensis*. It occupies sub‐ to intertidal habitats, but occurs in estuarine habitats during high tide (McGaw, 2005). It preys on barnacles, amphipods, intertidal invertebrates, and smaller crabs, including *Hemigrapsus* spp.

*Hemigrapsus oregonensis* and *Cancer productus* crabs were collected from a single location along an estuarine shoreline in Netarts Bay, Oregon (45°24′51.21″N, 123°56′4.38″W), on June 15, 2015. *Cancer productus* were caught using crab traps deployed at high tide, while *H. oregonensis* were hand captured along the shoreline. Both species were transported in chilled seawater to the laboratory at Portland State University. Upon arrival, *H. oregonensis* (*n* = 90) were sorted, measured, and randomly distributed into 30 housing tanks (~64 L, three crabs in each: one large dominant male, one small female, and one small male). *Cancer productus* (*n* = 15) were housed in three designated holding tanks (~120 L, five crabs in each) not dosed with fluoxetine.

Housing tanks were designed to simulate the estuarine conditions from which the *H. oregonesis* were collected. Each tank was filled with sand (500 g) and small pebbles (500 g) for burrowing substrate and one large rock (600–750 g) to hide under (Figure 1a). Each housing tank had an independent water chilling and filtration system (Aquatic Enterprises). Seawater was prepared using Instant Ocean and deionized water, and salinity and temperature were maintained at 35 PSU and 16.0°C to replicate conditions at the collection site. Light cycle conditions were maintained at 10 hr of dark and 14 hr of daylight.

Tanks were assembled on three racks (10 per rack) with sides blacked out with plastic lining to maintain behavioral isolation (see Figure 1b). Each tank contained three *H. oregonensis*: one large dominant male (hereafter, Dom M: mean carapace width (CW) ± *SE* = 25.54 ± 0.42 mm; mean wet biomass ± *SE* = 9.3 ± 1.4 g), one small female (hereafter, Sub F: CW = 19.25 ± 0.74 mm; 3.6 ± 1.5 g), and one small male (hereafter, Sub M: CW = 21.29 ± 0.65 mm; 4.97 ± 0.97 g). Mean carapace width and wet biomass did not significantly differ among treatment levels or tanks (two‐way ANOVA, *p *≥* *.4 in both cases). Crab densities (3.0/30 cm^2^) were lower than *H. oregonensis* densities at the collection site (up to 20 crabs/50 cm^2^; J. R. Peters, personal observation). However, we kept crab densities low to allow enough space in the tanks for escape from the much larger *C. productus* (range: 100 to 150 mm CW) during predator trials (see Figure 1a–c).

Animals were allowed to acclimate to aquarium habitats and laboratory conditions for 2 weeks before the behavioral study began. During the acclimation period, crab health and condition were monitored. A total of eight *H. oregonensis* died during acclimation (which were dispersed across treatments: 3 (30 ng/L), 2 (3 ng/L), and 3 (Control) and were immediately replaced with one of the extra crabs of the same sex and size class from the original collection. Every 2 days, *H. oregonensis* were fed a diet of squid or shrimp pieces. In addition, *H. oregonensis* regularly grazed microalgae from rocks and sediment and filter fed by rapidly beating their third maxillipeds near their mouth. *C. productus* were fed squid every 2 days.

Fluoxetine treatment concentrations were reached using separate dosing solutions prepared through serial dilution of an original stock solution of 1.0 mg/ml fluoxetine hydrochloride (Sigma‐Aldrich) dissolved in nanopure water. Every 10 days, tanks were dosed by adding 193 μl of dosing solution into each tank bringing the concentrations up to 3 and 30 ng/L. Controls without fluoxetine received 193 μl of nanopure water. Each fluoxetine treatment group (Controls, 3, and 30 ng/L) had 10 replicate tanks. To reduce buildup of nitrogenous wastes, 20% of the seawater was replaced with fresh seawater every 20 days, followed by another dosing of fluoxetine.

### 2.2 Behavioral study

Our behavioral study began June 29, 2015, and trials were conducted over a 9‐week fluoxetine exposure period. Each week, we conducted four trials with and without a predator observed during the day and night (i.e., day − predator, day + predator, night −predator, night + predator). During predator trials, *C. productus* were added directly to *H. oregonensis* housing tanks, occupying the same space for the hour‐long trial (Figure 1c). Using ethograms, observers recorded behavioral data during hour‐long trials. Recorded behaviors were organized by category: still, mobile, foraging, and species interactions. Still behaviors were when a crab remained buried or still. Mobile behaviors included the following: walking, digging, and moving in place. Foraging behaviors included crabs actively probing or eating food. Species interactions included agonistic, social, and predator avoidance behaviors. Agonistic behaviors were defined as aggressive interactions between conspecifics such as fighting or charging one another. During predator trials, we recorded predator avoidance behaviors, where *H. oregonensis* did or did not move away from *C. productus*. We also recorded the number of *H. oregonensis* killed by *C. productus*.

Behavioral acts per tank were recorded via instantaneous scanning at 5 min intervals for 1 hr. Scans were spaced at 5 min intervals to allow a reasonable amount of time to account for changes in behaviors over the duration of trial. Scans lasted 30 s and were standardized with a timer, allowing the observer to record acts of three individuals in each tank. Individual crabs were identified based on morphological differences (i.e., carapace and claw size). Thus, a total of 12 possible behavioral acts were recorded during each scan of an animal during the hour period. Day trials were conducted from 10:00 to 11:00 a.m., and night trials were conducted from 7:00 to 8:00 p.m. During night trials, we used red LED lights to record observations to minimize the effects of visible light wavelengths on nocturnal behaviors (Figure 1d). Trials without predators (both day and night) preceded trials with predators by 24 hr. Because the same crabs were being observed over the 9‐week study, we allowed 3 days in between predator trials each week to allow crabs to recuperate from stress. All trials were conducted from June 29 to August 27, 2015.

During the exposure study (60 days), and across all three fluoxetine treatments, 31 crabs perished either through predation by *C. productus* during trials (25) or through conflicts between conspecifics (6), in which case each was immediately replaced by an individual of the same size class and sex. Replacement was necessary in order to maintain consistency in species interactions among three individuals across all treatments, although it likely introduced an artifact of fluoxetine‐treated crabs interacting differently with new unexposed crabs. However, we felt that it was more important to keep the number of crabs consistent in each tank during trials. We excluded replacement crabs from subsequent analyses because our questions were centered on fluoxetine exposure.

### 2.3 Statistical analyses

Our analyses were based on counts of behavioral acts recorded during each trial.

We a priori grouped behaviors that we considered high‐risk (i.e., mobile, foraging, and species interactions) and low‐risk (i.e., remaining buried or still) to calculate the proportion of risk behaviors during weekly trials. Because the risk behavior response variable was proportional with a discrete outcome of 0–1, we used mixed logit models to test the probability of crabs successfully exhibiting risk behaviors during the trials. As our experiment was a repeated measures design, we fitted each model with random intercepts for tanks and trials to account for correlations in crab behaviors associated with sharing the same tank and over successive trials. Model fixed factors included the following: fluoxetine concentrations (Control, 3, and 30 ng/L), crab sex (Dom M, Sub F, Sub M), time (day, night), trial type (predator, no predator), and the exposure period (in weeks).

For hypothesis testing, we used likelihood ratio tests (LRT) with chi‐square test statistics to compare null models with each main term through stepwise selection of the best‐fit model based on Akaike Information Criterion (AIC). If main terms significantly improved the model fit, they were included in the full model. Because our hypotheses centered on the interaction between experimental factors and fluoxetine treatment, we used LRTs to test each interaction with the full model, following the same stepwise procedure for main terms. Interactions that were significant were included in the final best‐fit model. Model assumptions of normality and homoscedasticity were assessed through visual inspection of the residuals. Post hoc contrasts between experimental factors were then tested for significance with a Tukey HSD test using the lsmeans package (Lenth, 2016).

We pooled counts of species interaction behaviors (i.e., agonistic and active predator escape) into three exposure periods (1–3, 4–6, and 7–9 weeks) because they did not occur in every trial. We then compared these counts of agonistic and predator escape behaviors among fluoxetine treatments and experimental conditions using a generalized mixed model (GLMM) fitted with a Poisson distribution. The agonistic and predator escape GLMMs included the same fixed factors and random intercepts as the risk behavior mixed logit model. However, predator escape behaviors were restricted to trials with predators only; therefore, this GLMM did not include trial type as a factor. Hypothesis testing was conducted following the LRT framework outlined above.

Assumptions of normality and homoscedasticity for all GLMMs were assessed through visual interpretations of the residuals. We also checked GLMMs for overdispersion by calculating the ratio of residual deviance to residual degrees of freedom. To account for overdispersion, we added an observation‐level random effect to avoid biased parameter estimates. All GLMM analyses were performed using the glmer function in the lme4 package (Bates, Machler, Bolker, & Walker, 2015) in R (R Core Team, 2016).

## 3 RESULTS

**Figure 2:**
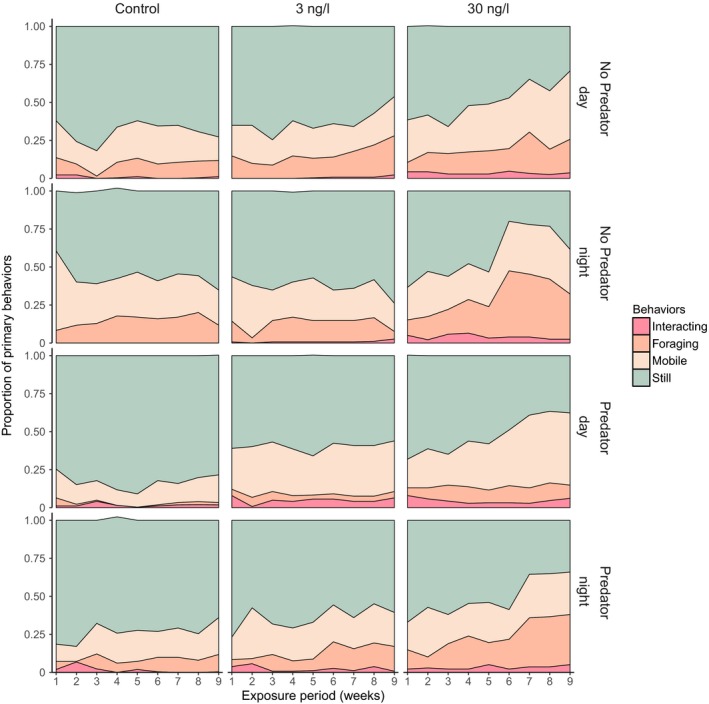
Weekly mean proportions of all crab behavioral categories over the duration of the study. Total proportions separated by different fluoxetine treatments during predator trials observed at day and night

Fluoxetine greatly affected *H. oregonensis* behaviors (mixed logit model; LRT, χ^2^ (2) = 11.89, *p *<* *.01), as those considered high‐risk (mobile, foraging, and species interactions) increased in treated crabs relative to controls over the course of the study (Figure 2). This increase in risk behaviors with prolonged exposure was consistent among crabs treated with 30 ng/L fluoxetine, irrespective of predator presence or time of day. However, crabs exposed to 3 ng/L fluoxetine did not follow this trend, and instead behaved more like control crabs. Control crabs were predominately still during the trials; however, they exhibited more active behaviors at night, particularly when *C. productus* was not present (Figure 2 and Appendix S1–S3). Predator presence had a strong effect on crab behaviors (LRT), χ^2^ (1) = 6.47, *p *=* *.01), decreasing the probability of (diurnal—nocturnal) risk activity in control crabs to 0.15–0.27 (i.e., remaining still 85%–73% of the time). The predator effect on risk behaviors decreased with increasing fluoxetine concentration: 3 ng/L (0.35–0.40), 30 ng/L (0.47–0.49). Activity among controls though more variable within the first few weeks remained fairly consistent throughout the 9‐week study (Figure 2). Crabs treated with fluoxetine had more variable behavioral patterns, although those exposed to 3 ng/L were more consistent over time. Crabs in the 30 ng/L treatment group exhibited considerable changes in behavioral patterns during the study, where risk behaviors were more prominent with increased exposure (Figure 2).

### 3.1 Risk behaviors

**Figure 3:**
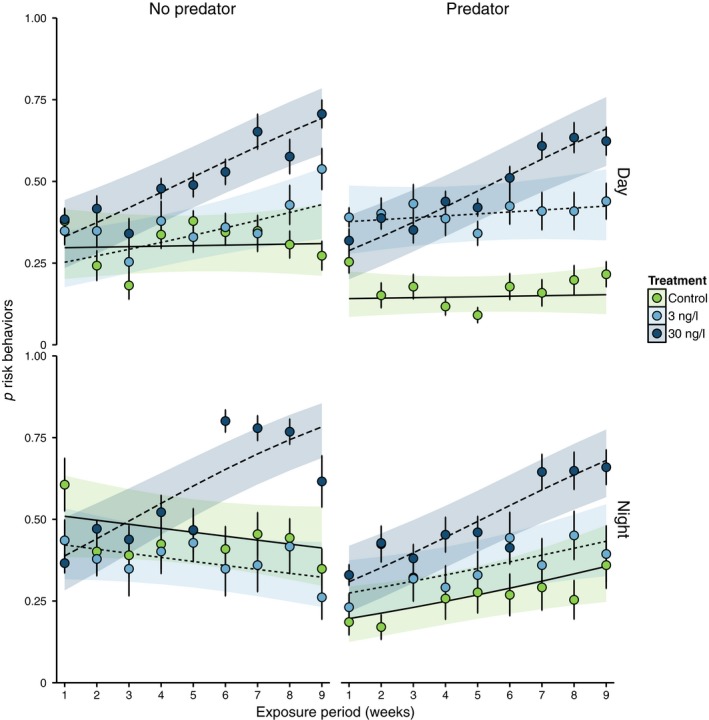
Weekly mean observed proportions of *Hemigrapsus oregonensis* risk behaviors under different fluoxetine treatments. Error bars depict standard error of the means. Lines represent mixed logit model‐predicted probabilities for each fluoxetine treatment with bands depicting 95% confidence intervals. Values separated by trials with and without predators observed at day and night

Risk behavior data were best‐fit by a mixed logit model with two significant 3‐way interactions (fluoxetine treatment × trial type × time) and (fluoxetine treatment × time × exposure period) as well as their respective main terms (LRT, χ^2^ (10) = 125.28, *p *<* *.001); indicating that the effects of fluoxetine on these behaviors are mediated by length of exposure, presence of a predator, and time of day. Crab sex and size class did not significantly improve the model fit (LRT, χ^2^ (4) = 1.60, *p = *.12) and were therefore dropped from the final risk behavior model. The final model was used to predict probabilities of *H. oregonensis* exhibiting risk behaviors based on observed proportions (Figure 3).

*Hemigrapsus oregonensis* risk behaviors were affected by fluoxetine exposure, mediated by an interaction with predator presence and time of day (LRT, χ^2^ (7) = 71.41, *p *<* *.001). This interaction was due to an increased probability of crabs exhibiting risk behaviors among the 30 ng/L treatment group (range of predicted probabilities = 0.47–0.60) across the combination of trial types (no predator/predator × day/night). In contrast, the probabilities of crabs in 3 ng/L and control groups exhibiting risk behaviors were (0.33–0.40) and (0.15–0.45), respectively (Figure 3). Crabs in control groups were least likely to take risks during a daytime predator trial (mean predicted probability = 0.15), remaining still or buried 85% of the time (Figures 2 and 3). Control crabs were twice as likely (0.30) to take risks during the daytime without a predator; however, they remained still or buried 70% of the time, while at night predator presence reduced risk behaviors from 0.45 to 0.27 (Figures 2 and 3). Conversely, crabs exposed to 3 ng/L fluoxetine did not reduce their daytime risk behaviors during a predator trial (0.40), which was even a slight increase from trials without a predator (0.34). They also exhibited a similar amount of risk behaviors during nighttime predator (0.35) and no predator trials (0.37). Crabs in the 30 ng/L group had the highest probability of exhibiting risk behaviors: 0.51 without predators and 0.47 with predators during the day, and 0.60 without predators and 0.49 with predators during the night.

The effect of fluoxetine on *H. oregonensis* risk behaviors also depended on the length of exposure and by the time of day (LRT, χ^2^ (7) = 71.41, *p *<* *.001). This 3‐way interaction was driven by differences in observed risk behaviors between day and night among the fluoxetine treatment groups and how those patterns changed over time (Figures 2 and 3). In the control group, there was a consistent trend of low activity during the day and increased activity at night (Figures 2 and 3, Appendix S1–S3). However, this pattern did not hold for fluoxetine‐treated crabs, as both the 3 ng/L and 30 ng/L groups were just as likely to be active during the day as they were at night (Figures 2 and 3). Yet over the course of the study, crabs in the 30 ng/L treatment group significantly increased their risk behaviors from 0.28–0.41 in week 1 to 0.67–0.77 by week 9. Risk behaviors were more consistent between week 1 and week 9 for the 3 ng/L (0.28–0.36 in week 1 and 0.36–0.47 by week 9) and control groups (0.15–0.42 in week 1 and 0.15–0.49 by week 9).

### 3.2 Species interactions

Fluoxetine had a strong effect on *H. oregonensis* agonistic behaviors (GLMM; LRT, χ^2^ (2) = 199.33, *p *<* *.001, Table 1). Crabs exposed to 30 ng/L of fluoxetine were 7.72 times more likely (C.I. = 3.52–16.9) to engage in agonistic behaviors than crabs in control groups. Sex and exposure periods were not important factors on their own (GLMM; LRT, χ^2^ = 3.23, 2.71, *df* = 2, 4, *p *≥* *.2, respectively, Table 1) but their interactions with fluoxetine, along with the interactions among all other experimental factors contributed to the best model fit (GLMM; LRT, χ^2^ = .37, *df* = 12, 22, *p *<* *.001).

**Table 1:** Counts of agonistic behaviors within pooled exposure periods. Percent of total counts were calculated by trial type (i.e., Day/Night and (+/−) Predator). Results from likelihood ratio test, LRT, comparing counts of agonistic behaviors between interaction and null models, fitted with a Poisson distribution^a^

Time	(+/−) Predator	Treatment	Exposure			Total	% of Total
			Weeks (1–3)	Weeks (4–6)	Weeks (7–9)		
Day	−	Control	4	5	8	17	13.6
		3 ng/L	0	5	11	16	12.8
		30 ng/L	45	29	18	92	73.6
	+	Control	2	0	0	2	3.7
		3 ng/L	11	13	9	33	62.3
		30 ng/L	12	3	3	18	34.0
Night	−	Control	8	12	8	28	16.4
		3 ng/L	4	6	14	24	14.0
		30 ng/L	45	45	29	119	69.6
	+	Control	0	5	2	7	8.1
		3 ng/L	7	10	6	23	26.7
		30 ng/L	14	21	21	56	65.1

a Poisson generalized mixed model, LRT: χ^2^ (8) = 66.77, *p *<* *.001.

Time of day had the strongest effect on active predator escape behaviors (GLMM; LRT, χ^2^ (1) = 68.77, *p *<* *.001, Table 2). Counts of active predator escape were higher during the day than at night. Fluoxetine treatment also had a strong effect on predator escape behaviors (GLMM; LRT, χ^2^ (2) = 16.49, *p *<* *.001), with more counts of escape in 3 ng/L (168) and 30 ng/L (157) than control groups (104) over the course of the study. Sex and size class was not an important factor in driving predator escape patterns (GLMM; LRT, χ^2^ (2) = 3.90, *p *=* *.14).

**Table 2:** Counts of active predator escape within pooled exposure periods. Percent of total counts were separated by day and night trials. Results from likelihood ratio test, LRT, comparing counts of escape behaviors between interaction and null models, fitted with a Poisson distribution^a^

Time	Treatment	Exposure			Total	% of Total
		Weeks (1–3)	Weeks (4–6)	Weeks (7–9)		
Day	Control	28	18	13	59	19.7
	3 ng/L	28	43	46	117	39.0
	30 ng/L	50	41	33	124	41.3
Night	Control	35	3	7	45	34.9
	3 ng/L	28	17	6	51	39.5
	30 ng/L	9	16	8	33	25.6

a Poisson generalized mixed model, LRT: χ^2^ (8) = 44.15, *p *<* *.001.

Overall, 31 crabs perished during the study: 25 were killed by *C. productus,* and six were killed through fighting with conspecifics. Of those killed, 13 (42%) were in the 30 ng/L group (nine by predator, four by conspecifics), 10 (32%) in the 3 ng/L group (eight by predator, two by conspecifics), and eight (26%) in the control group (eight by predator, 0 by conspecifics).

## 4 DISCUSSION

In the presence of predators, prey will often modify their behaviors to balance the risk of mortality with the reward of accessing food, mates, or other resources (Catano et al., 2016; Sih, Cote, Evans, Fogarty, & Pruitt, 2012; Snell‐Rood, 2013). Prey may reduce their activity levels, utilize defenses, or seek refuge when they perceive the risk to be high (Lima & Dill, 1990; Lindberg, 1980). Our results indicate that higher concentrations of fluoxetine stimulate crab activity levels and reduce their inhibition to predator threats. The alterations we observed in their diurnal and nocturnal behaviors may place crabs inhabiting harbors or estuaries contaminated with fluoxetine at greater risk of predation and mortality.

We designed this experiment to simulate estuarine conditions in the laboratory, reducing variation among tanks by maintaining identical abiotic conditions (e.g., light, temperature, and salinity) and habitat substrate (e.g., rocks, gravel, and sand) across treatments. Therefore, we propose that the differences in crab behavior reported here were not attributable to experimental artifacts. Additionally, we believe any learned tolerance of the predator was minimal because (1) we allowed for sufficient time between predator trials; (2) we did not preclude *C. productus* from predating on *H. oregonensis* during the trials; and (3) predator induced mortality did not decline over time. Further, our observed proportions of crab active and predator avoidance behaviors in controls did not change significantly during the study.

Our results suggest fluoxetine affected crab diurnal and nocturnal behaviors, making them more prone to predation risk. Like other crabs, *H. oregonensis* are photonegative, emerging primarily at night to forage to avoid encounters with predators. We expected higher activity among all crabs during night trials. However, crabs exposed to 30 ng/L of fluoxetine exhibited substantially more activity during the day than controls, disrupting the normal daytime patterns of staying hidden or buried. Crabs exposed to this amount of fluoxetine over an extended period are inherently more prone to predation risk. We also found that extended exposure to fluoxetine exacerbated the effect on risk behaviors, as crabs in the 30 ng/L group were most likely to engage in risk activity following 7–9 weeks of exposure. Perhaps this is due to bioconcentration of the drug in animal tissue as fluoxetine hydrochloride is a lipophilic compound (Kreke & Dietrich, 2008). Interestingly, there was little difference between diurnal and nocturnal activity levels in crabs exposed to 3 ng/L of fluoxetine. Perhaps photoperiod was not as important for regulating activity in this treatment group or exposure to fluoxetine increased diurnal activity enough to cause these behaviors to level out over time.

Serotonin and serotonin analogs have been shown to alter agonistic behaviors (McPhee & Wilkens, 1989; Tierney & Mangiamele, 2001) and activity levels (Fong & Ford, 2014; Perez‐Campos, Rodriguez‐Canul, Perez‐Vega, Gonzalez‐Salas, & Guillen‐Hernandez, 2012) in crustaceans. Fluoxetine concentrations ≥120 μg/L caused a stimulation of locomotor behavior in the crab *Carcinus maenas* (Mesquita et al., 2011). We found similar increases in mobile behaviors in *H. oregonensis* exposed to only 30 ng/L of fluoxetine. In *Chasmagnathus* crabs, Pedetta, Kaczer, and Maldonado (2010) modulated individual aggressiveness via manipulation of serotonin and octopamine levels, where aggressiveness increased and decreased with the addition of the respective hormone. Our results demonstrate similar effects in *H. oregonensis*. Perhaps fluoxetine, through modulation of serotonin levels, stimulates crab activity levels and drives aggressive behaviors. Fluoxetine's effect on serotonin levels appears to increase boldness and potentially other risk behaviors as studies on other species have suggested (Dzieweczynski & Hebert, 2012; Fong & Ford, 2014; Mesquita et al., 2011; Pedetta et al., 2010; Tierney & Mangiamele, 2001).

Fluoxetine is one of the most widely used antidepressants in the world (Ankley et al., 2007; Brooks et al., 2003) and a large amount of research has documented its occurrence in aquatic (Bringolf et al., 2010; Corcoran et al., 2010; Kwon & Armbrust, 2006; Ramirez et al., 2009) and marine (Kreke & Dietrich, 2008; Vasskog et al., 2008) environments. With growing human populations in coastal zones, increasing use of antidepressants like fluoxetine is expected, suggesting higher future concentrations in the marine environment. Our results demonstrate how pharmaceuticals affect species behaviors and their interactions. Brodin et al. (2014) nicely summarized several ecologically important behavioral traits for assessing sublethal effects of pharmaceutical exposure, and potential direct or indirect ecological effects. These behavioral traits include the following: activity, aggression, boldness, exploration, and sociality. Each of these traits lead to direct ecological effects such as dispersal/migration, feeding rates, mating success, parental care, and predator avoidance—and changes in these traits have consequences for individual fitness (Gross, 2005). These direct effects can be linked to differences in community structure, cross‐boundary effects, ecosystem function, feedback loops, population dynamics, and trophic cascades. Anthropogenic impacts to coastal systems such as ocean acidification and rising temperatures have been identified as significant environmental stressors, altering much of the aforementioned ecosystem processes (Dodd et al., 2015; Fukunaga et al., 2010; Munday et al., 2009). As pharmaceuticals affect many of the same processes through similar mechanisms, they warrant consideration as an important anthropogenic stressor in need of further research.

Estuarine and coastal organisms are exposed to whole suites of contaminants, many of which (e.g., sertraline (Effexor^®^; Bossus et al., 2014), carbamazepine (Tegretol^®^; Martin‐Diaz et al. 2009)) have known negative effects on aquatic and marine organisms (e.g., Fong & Molnar, 2008; Metcalfe et al., 2010; Meredith‐Williams et al., 2012; Gaw et al., 2014). Our study and others have assessed the effects of single pharmaceuticals on animal behavior and their potential to alter species interactions (Bossus et al., 2014; Gaworecki & Klaine, 2008; Hazelton et al., 2013; Piggott, Baldwin, Dissanayake, & Sloman, 2007). Yet, additional studies examining the effects of multiple compounds are warranted to understand interactive and cumulative effects on organisms and ecosystems (Brausch et al., 2012; Brodin et al., 2014). Furthermore, studies that assess how pharmaceuticals interact with ocean acidification conditions would add to the growing field of multiple stressor research. To our knowledge, no studies have assessed ecosystem responses to pharmaceuticals or other emerging contaminants. That would be an important next step in understanding how these compounds may influence essential processes.

## CONFLICT OF INTEREST

None declared.

## AUTHOR CONTRIBUTIONS

JRP involved in conception of project, experimental design, collected the data, analyzed and interpreted the data, and drafted the article; EFG & CER involved in project development and critically revised the article; MR collected the data.
