# Supplementary material for: Prozac in the water: Chronic fluoxetine exposure and predation risk interact to shape behaviors in an estuarine crab
Source: Ecol Evol. 2017 Sep 30;7(21):9151–61. doi: 10.1002/ece3.3453 (PMC5677497; doi:10.1002/ece3.3453)

Appendix S1. Mean proportions of mobile behaviors separated by experimental factors. Error bars depict the standard error of observed proportions.


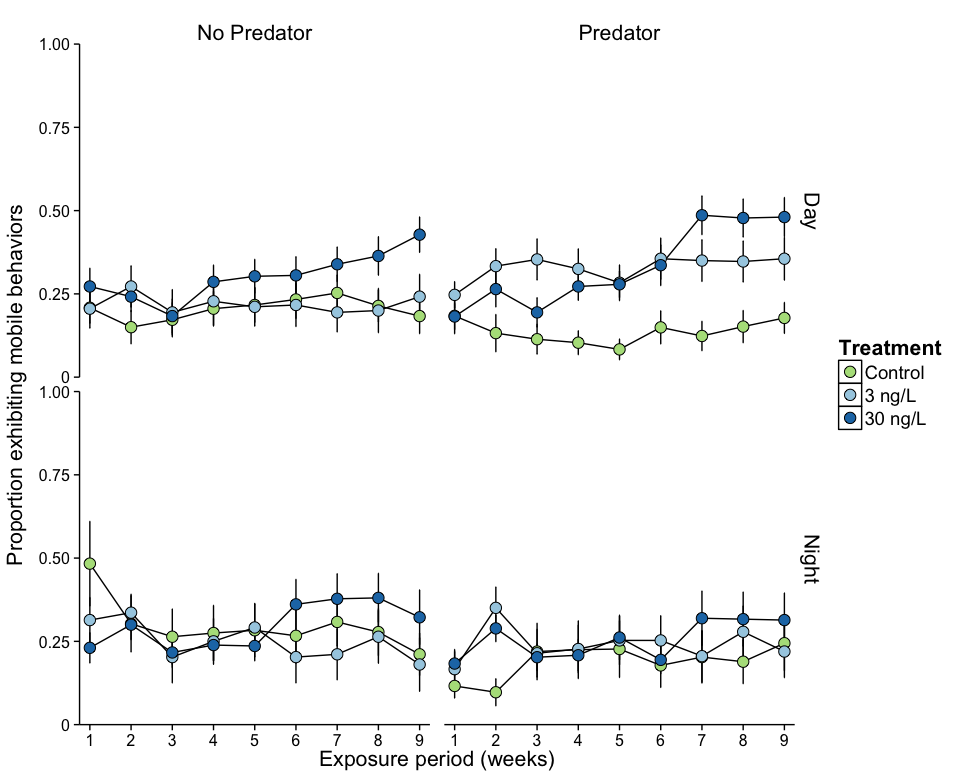


Appendix S2. Mean proportions of foraging behaviors separated by experimental factors. Error bars depict the standard error of observed proportions.


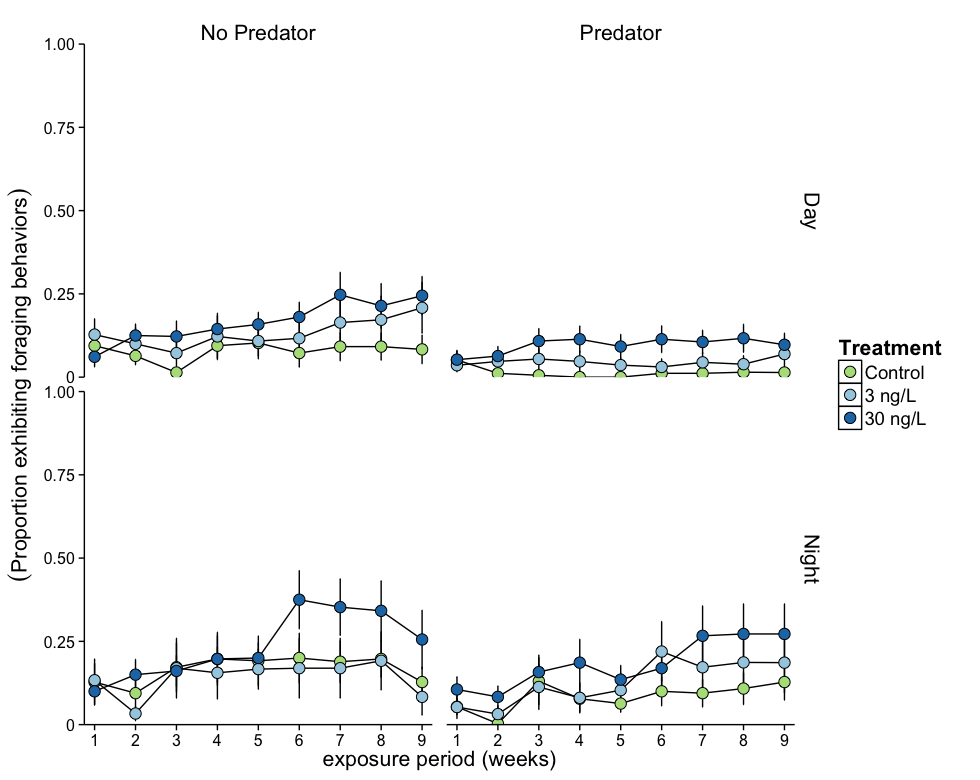


Appendix S3. Mean proportions of still behaviors separated by experimental factors. Error bars depict the standard error of observed proportions.


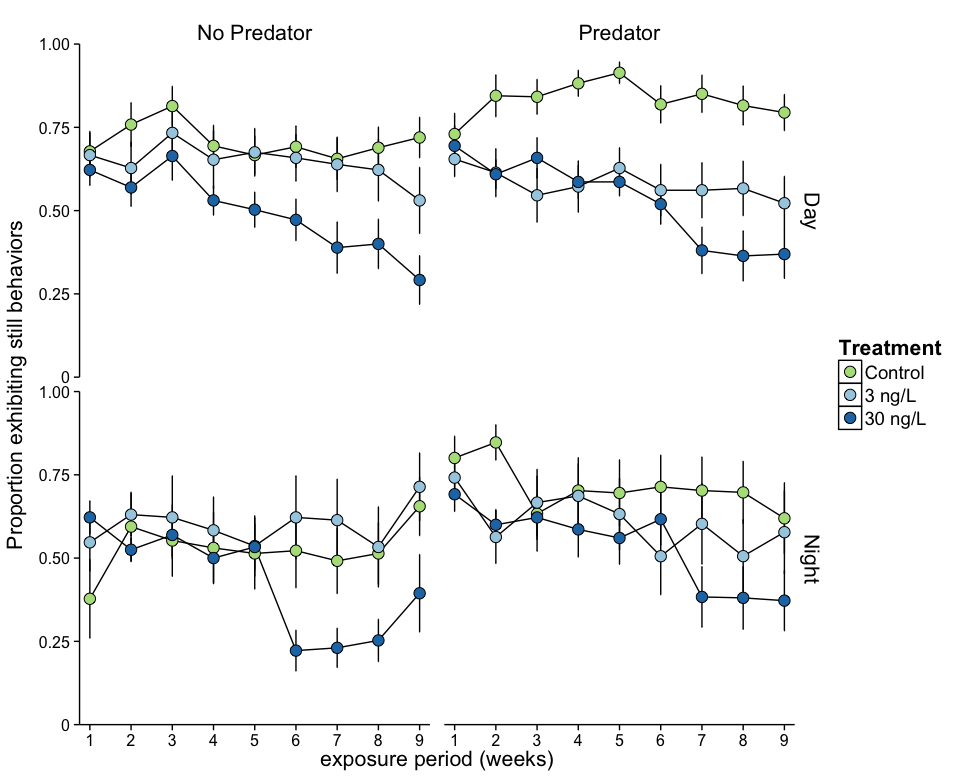

Supplement: Supplementary file 1 [file ECE3-7-9151-s001.docx]
